# Long non-coding RNA plasmacytoma variant translocation 1 and growth arrest specific 5 regulate each other in osteoarthritis to regulate the apoptosis of chondrocytes

**DOI:** 10.1080/21655979.2022.2063653

**Published:** 2022-06-15

**Authors:** Liquan Cai, Nianlai Huang, Xiaolu Zhang, Shiqiang Wu, Liangming Wang, Qingfeng Ke

**Affiliations:** Department of Trauma Orthopaedics, Joint Surgery, Donghai Hospital, The Second Affiliated Hospital of Fujian Medical University, Quanzhou City, Fujian Province, China

**Keywords:** Osteoarthritis, PVT1, GAS5, chondrocytes, apoptosis

## Abstract

Long non-coding RNA (lncRNA) plasmacytoma variant translocation 1 (PVT1) and growth arrest specific 5 (GAS5) have opposite functions in the apoptosis of chondrocytes, which are involved in the pathogenesis of osteoarthritis (OA). The opposite roles of PVT1 and GAS5 in OA may indicate the existence of crosstalk between them in OA. This study aimed to explore the possible interaction between PVT1 and GAS5 in OA. Accumulation of PVT1 and GAS5 in OA and control synovial fluid samples was measured by RT-qPCR. The interaction between PVT1 and GAS5 in chondrocytes was explored by overexpression experiments. Dual-luciferase reporter assay was performed to analyze the binding of PVT1 and GAS5 to each other’s promoter regions. Regulatory roles of PVT1 and GAS5 in the apoptosis of chondrocytes were studied with cell apoptosis assay. PVT1 was upregulated in OA, and GAS5 was downregulated in OA. An inverse correlation between PVT1 and GAS5 was observed across OA samples. Under lipopolysaccharides (LPS) treatment, PVT1 was upregulated and GAS5 was downregulated. Interestingly, PVT1 and GAS5 overexpression downregulated each other in chondrocytes. Cell apoptosis analysis showed that PVT1 overexpression promoted cell apoptosis, while GAS5 overexpression suppressed cell apoptosis induced by LPS. Co-transfection of PVT1 and GAS5 failed to significantly affect cell apoptosis. PVT1 and GAS5 directly bound to each other’s promoter regions. Our study characterized the interaction between PVT1 and GAS5 in OA. Their interaction regulated the apoptosis of chondrocytes, which play a critical role in OA. PVT1 and GAS5 may form a negative feedback loop in OA.

## Graphical abstract

**Figure:**
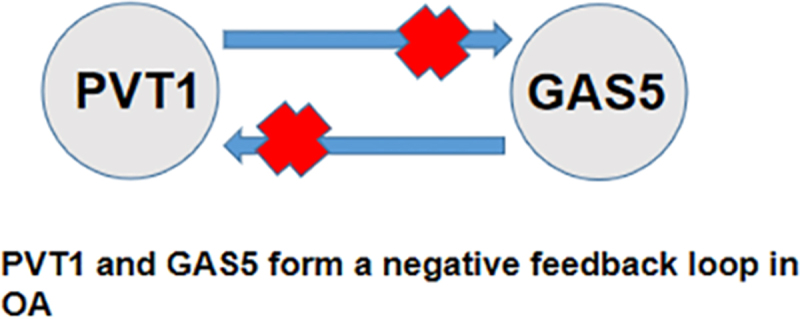

## Background

Osteoarthritis (OA), also known as the ‘wear and tear’ of the joints or degenerative joint disease, is a common joint disease caused by the breakdown of the underlying bone and joint cartilage [1]. Primary OA develops with aging, prior injury/trauma, and inflammatory arthritis [2] and affects more than 10% or even 30% of people older than 60 years [3]. No treatment approaches have been developed to cure OA, and most of the available treatments (except DMOA therapies) can only be applied to control symptoms, such as primarily pain [4,5]. To this end, OA treatment requires novel insights.

Studies on OA pathogenesis revealed the involvement of altered signaling pathways in the molecular pathogenesis of OA [6–8]. These molecular players may be targeted for OA therapy. Such targeted therapy may be developed to cure OA in many cases by regulating related gene expression [9]. Besides protein players, the development of OA also requires the involvement of long non-coding RNAs (lncRNAs) [10], which participate in the development of OA and other human diseases, such as cancers, heart diseases, and metabolic diseases, by regulating disease-related gene expression but not protein synthesis [11,12]. Hence, lncRNAs may serve as potential targets for OA treatment. However, our understanding of their functions is still limited. Previous studies have reported that lncRNAs plasmacytoma variant translocation 1 (PVT1) and growth arrest specific 5 (GAS5) have opposite functions in the apoptosis of chondrocytes [13,14], which play critical roles in OA development. PVT1 has been identified to promote NLRP3-mediated pyroptosis in septic acute kidney injury [15]. PVT1 was also demonstrated to accelerate LPS-induced viability and migration of cardiac fibroblasts [16]. Additionally, PVT1 has been revealed to sponge miR-488-3p to increase chondrocyte apoptosis in OA [13]. By contrast, GAS5 overexpression in ATDC5 chondrocytes has been reported to upregulate KLF2, thereby reversing LPS-induced apoptosis and inflammatory injury [14]. In addition, a recent study also proved that GAS5 was upregulated in OA serum and cartilage tissues and cells, and induced chondrocyte apoptosis [17]. The opposite role of PVT1 and GAS5 in chondrocyte apoptosis may indicate their potential interaction in OA. Thus, the study was carried out to analyze the interaction between PVT1 and GAS5 in OA and explore the involvement of their interaction in chondrocyte apoptosis.

## Methods

### Research subjects

The study was approved by the Ethics Committee of Donghai Hospital, the Second Affiliated Hospital of Fujian Medical University (Supplemental file 1). At this hospital, 62 OA patients (Kellgren and Lawrence system grade 4, 26 males and 36 females, 51–68 years, 59.3 ± 6.3 years) and 62 healthy controls (26 males and 36 females, 51–68 years, 59.2 ± 6.4 years) were enrolled from May 2017 to May 2019. The participants in the two groups showed similar age and gender distributions. OA patients were diagnosed by analyzing X-ray images and clinical signs. Healthy controls with normal physiological functions were enrolled after completing systemic physiological examinations (including joint analysis). None of the controls had a history of OA. Patients were excluded if they had other clinical disorders (severe infections, metabolic disorders, cancers) or were treated for any clinical disorders within 3 months before admission. All patients and controls signed the written informed consent.

### Synovial fluid preparation

Before the initiation of therapy, all patients were subjected to synovial fluid (about 1.0 ml) extraction from the affected sites (30 cases of knee and 32 cases of hip). In addition, the same amount of synovial fluid was also extracted from the knee (n = 30) and hip (n = 32) from healthy controls. In addition, joint fluid was also extracted from both patients and healthy controls using a syringe under local anesthesia. RNA samples were isolated from these synovial fluid samples immediately after extraction.

### Synovial tissues

A total of six fixed OA (grade 4) synovial tissue samples and four fixed control synovial tissue samples were obtained from the specimen library of our hospital. These synovial tissues were extracted from each operated knee during total knee arthroplasty. The OA samples were from two males and four females (55–65 years). The control samples had no pathological changes and were from one male and three females (59–66 years) who received knee joint surgery due to trauma. All participants agreed on the day before surgery to have their synovial tissues removed and used for research.

### Chondrocytes and cell transfections

Chondrocytes (402OA-05A, Sigma-Aldrich) derived from an OA patient were cultured in chondrocyte growth media (PromoCell) in an incubator with 95% humidity and 5% CO_2_ at 37°C. To study the effects of lipopolysaccharides (LPS) treatment on gene expression, cells were cultured in a medium supplemented with LPS (0, 1, 2, 4, 8, and 12 ng/ml) for 48 h before using [18].

PVT1 and GAS5 vectors were made with pcDNA3.1 (Invitrogen, USA) or pCDNA3.1-EGFP (Invitrogen, USA). The chondrocytes collected from passages 3–5 were transfected with either PVT1 or GAS5 vector (1 µg) or empty vector (1 µg, negative control experiment, NC) using lipofectamine 2000 (Invitrogen). GFP gene expression carried by pCDNA3.1-EGFP was observed under a fluorescence microscope.

### Dual luciferase reporter assay[19]

The promoter regions of PVT1 and GAS5 (from position −30 to −3000) were cloned into pGL3-Promoter Vector to establish luciferase vectors. The cells were then co-transfected with a PVT1 expression vector and GAS5 luciferase vector or GAS5 expression vector and PVT1 luciferase vector. The control group was the cells co-transfected with empty pGL3-Promoter Vector and PVT1 or GAS5 expression vector. Luciferase activity was determined at 48 h after transfection.

### RNA preparations

RNA isolation from all samples was done with RNAzol (Sigma-Aldrich). DNA contamination was removed using DNase I.

### RT-qPCR

RNA samples were reverse transcribed into cDNA samples using SuperScript™ IV First-Strand Synthesis System (Thermo Fisher) and used in qPCRs to analyze the accumulation of PVT1 and GAS5 with 18S rRNA internal control. Ct values were subjected to 2^−ΔΔCt^ normalization [20]. It is worth noting that similar results were obtained using GAPDH as an internal control.

### Apoptosis assay

The regulatory roles of PVT1 and GAS5 in chondrocyte apoptosis were explored using cell apoptosis assay. Chondrocytes were harvested after digestion with collagenase type II at 48 h post-transfection, further incubated in media containing 12 ng/ml LPS for 48 h, and stained with Annexin-V FITC and propidium iodide (PI). Finally, the apoptotic cells were separated using FACS Calibur instrument. In each experiment, 1 × 10^6^ cells were included.

### Western blot assay

Total proteins were extracted from *in vitro* cultured chondrocytes using Protein Extraction Kit (Novus Biologicals). After denaturation (boiling water for 10 min), proteins were separated on 10% SDS-PAGE gels and transferred onto PVDF membranes. The membranes were incubated first with rabbit anti-human Caspase-3 (Abcam, 1:1200), Bax (Abcam, 1:1200), and GAPDH (Abcam, 1:1400) for 14 h at 4°C, and then with HRP-labeled goat anti-rabbit secondary antibody (MyBioSource, 1:1200). Signals were developed using ECL. Caspase-3 and Bax proteins were included in this study to reflect cell apoptosis.

### Statistical analysis

Differences between the two groups and among multiple groups were compared using the Mann–Whitney test and the Kruskal–Wallis test, respectively. Correlations were analyzed by linear regression. P < 0.05 was considered statistically significant.

## Results

### PVT1 and GAS5 expression alteration was observed in OA synovial fluid cells

**Figure 1.**
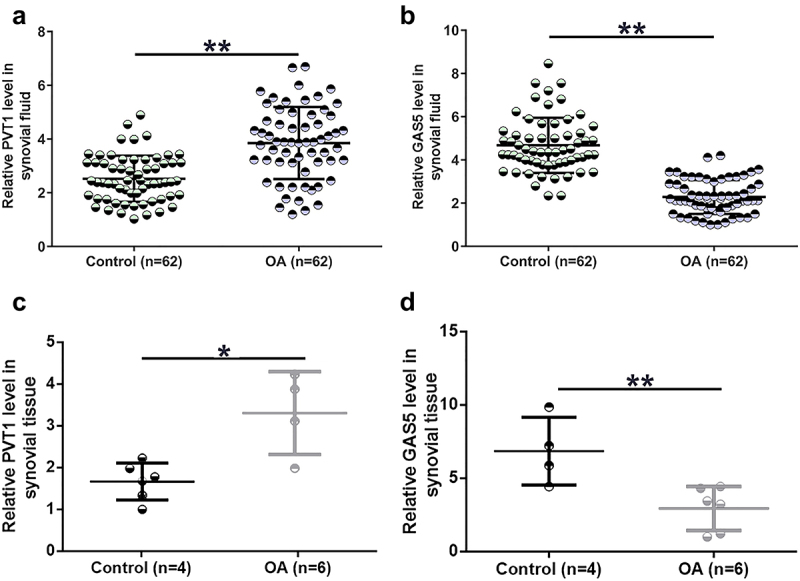
PVT1 and GAS5 expression alterations were observed in OA synovial fluid cells. PVT1 (a) and GAS5 (b) accumulation in synovial fluid samples from both OA patients (n = 62) and controls (n = 62) and expression of PVT1 (c) and GAS5 (d) in synovial tissue samples from both OA patients (n = 6) and controls (n = 4) were determined by RT-qPCR. The three lines represent 25%, median, and 75% values, respectively. *: p < 0.05; **: p < 0.01.

Gene expression determines function. To this end, expression levels of both PVT1 and GAS5 in OA synovial fluid and synovial tissues collected from both OA patients and controls were determined using RT-qPCR. PVT1 was accumulated to high levels in OA synovial fluid compared to the controls (Figure 1a, p < 0.01). In contrast, GAS5 level in synovial fluid was significantly lower in the OA group than in the control group (Figure 1b, p < 0.01). Consistently, PVT1 expression level in synovial tissues was also significantly higher in the OA group than in the control group (Figure 1c, p < 0.05). In addition, GAS5 expression level in synovial tissues was significantly lower in OA (Figure 1d, p < 0.01). These results suggest that altered expression of PVT1 and GAS5 may participate in OA.

### The correlation of PVT1 to GAS5

**Figure 2.**
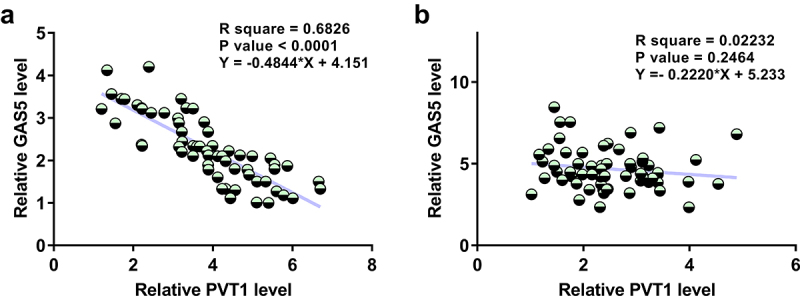
PVT1 and GAS5 were inversely correlated only in OA patients but not in healthy controls. The correlations of PVT1 to GAS5 across OA (a) and control (b) synovial fluid samples were studied with linear regression.

A close correlation may indicate a potential interaction. To this end, the correlations between PVT1 and GAS5 across OA and control synovial fluid samples were analyzed by linear regression analysis. It was observed that PVT1 and GAS5 were inversely and significantly correlated across synovial fluid samples from OA patients (Figure 2a, p < 0.0001) but not from healthy controls (Figure 2b, p = 0.2464). Therefore, PVT1 and GAS5 may interact with each other in OA.

### The role of LPS in PVT1 and GAS5 expression in chondrocytes

**Figure 3.**
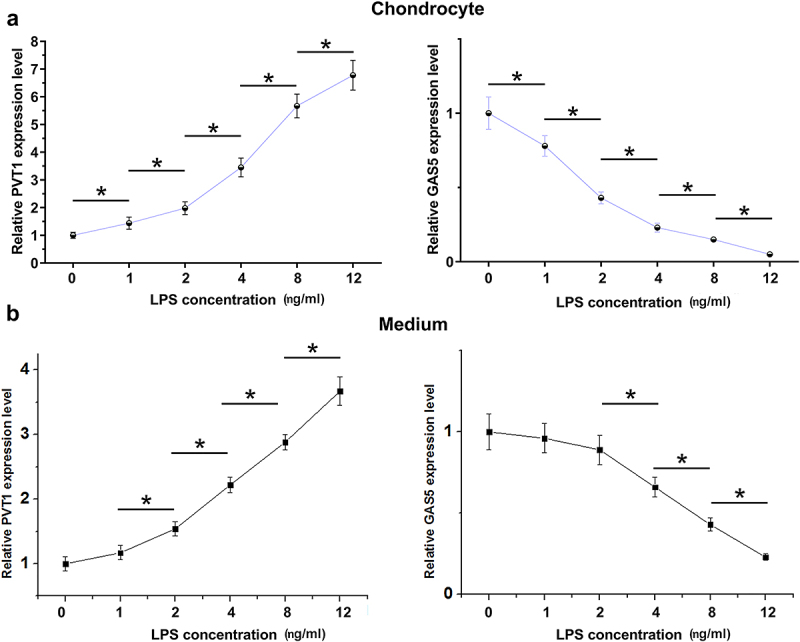
Regulatory role of LPS in PVT1 and GAS5 accumulation in chondrocytes. Chondrocytes were cultured in media supplemented with LPS (0, 1, 2, 4, 8, and 12 ng/ml) for 48 h, followed by determination of PVT1 and GAS5 in both chondrocytes (a) and cell culture medium (b). *: p < 0.05.

LPS-induced inflammation may be involved in OA. To this end, the cells were cultured in media supplemented with LPS (0, 1, 2, 4, 8, and 12 ng/ml) for 48 h. The cells were then collected, and PVT1 and GAS5 expression levels in chondrocytes and medium were determined by RT-qPCR. LPS treatment increased PVT1 accumulation and decreased GAS5 accumulation in a dose-dependent manner in both chondrocytes (Figure 3a, p < 0.05) and media (Figure 3b, p < 0.05). Therefore, PVT1 and GAS5 expression in chondrocytes and secretion into media can be regulated by LPS.

### PVT1 and GAS5 negatively regulated each other in chondrocytes

**Figure 4.**
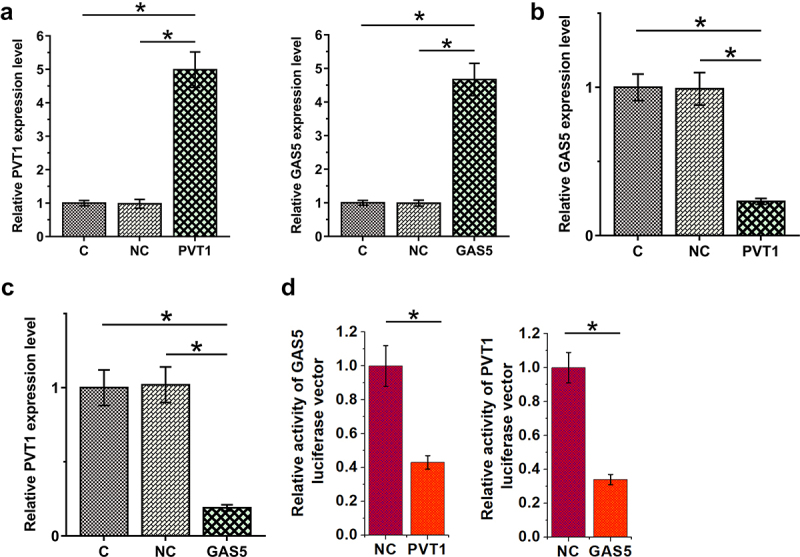
PVT1 and GAS5 negatively regulate each other in chondrocytes. Chondrocytes overexpressed PVT1 or GAS5 (a). The regulatory role of PVT1 in GAS5 accumulation (b) and the regulatory role of GAS5 in PVT1 accumulation (c) were studied with RT-qPCR. Dual-luciferase reporter assay was performed to analyze the binding of PVT1 and GAS5 to each other’s promoter regions (d). *: p < 0.05.

To study the interaction between PVT1 and GAS5 in OA, chondrocytes were transfected with PVT1 or GAS5 expression vectors and cells were harvested 24 h after transfection for subsequent experiments. The transfection rates were all above 90% (Supplemental file 2). The overexpression of both PVT1 and GAS5 was confirmed by RT-qPCR at 48 h after transfections (Figure 4a, p < 0.05). In addition, PVT1 overexpression downregulated GAS5 (Figure 4b, p < 0.05), and GAS5 also decreased PVT1 accumulation (Figure 4c, p < 0.05). Dual-luciferase reporter assay was performed to analyze the binding of PVT1 and GAS5 to each other’s promoter regions. Compared to the control group, PVT1 significantly reduced the luciferase activity of GAS5 luciferase vector. Similarly, GAS5 also significantly reduced the luciferase activity of PVT1 luciferase vector (Figure 4d). Therefore, PVT1 and GAS5 may form a negative feedback loop in chondrocytes by binding to each other’s promoters.

### The negative feedback loop formed between PVT1 and GAS5 regulates chondrocyte apoptosis induced by LPS

**Figure 5.**
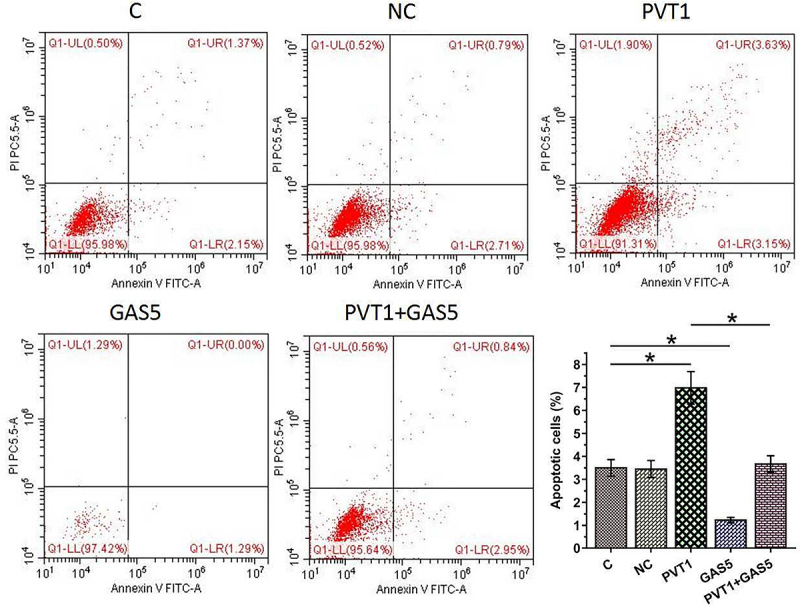
The negative feedback loop formed by PVT1 and GAS5 regulates chondrocyte apoptosis induced by LPS. The roles of PVT1 and GAS5 in regulating chondrocyte apoptosis induced by LPS (12 ng/ml) were explored by cell apoptosis assay. *: p < 0.05.

**Figure 6.**
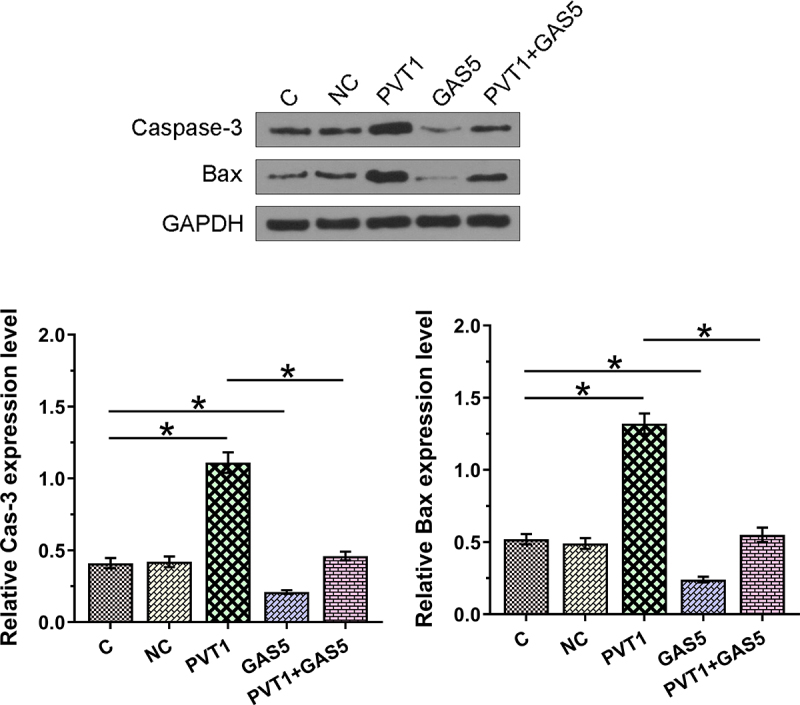
The negative feedback loop formed by PVT1 and GAS5 regulates chondrocyte apoptosis relative protein expression induced by LPS. The roles of PVT1 and GAS5 in regulating Cas-3 and Bax expression induced by LPS (12 ng/ml) were explored by western blot assay. *: p < 0.05.

Chondrocyte apoptosis affects the progression of OA. To this end, the roles of PVT1 and GAS5 in regulating chondrocytes apoptosis induced by LPS (12 ng/ml) were explored. Cell apoptosis analysis showed that PVT1 overexpression promoted cell apoptosis, while GAS5 overexpression suppressed cell apoptosis induced by LPS. In addition, the co-transfection of PVT1 and GAS5 failed to significantly affect cell apoptosis (Figure 5, p < 0.05). We further detected the expression of Cas-3 and Bax and found that PVT1 overexpression promoted the expression of both Cas-3 and Bax in cells induced by LPS, while GAS5 overexpression showed reversed effect (Figure 6, p < 0.05). Therefore, PVT1 and GAS5 may regulate each other to affect chondrocytes apoptosis in OA. Original western blot images are presented in supplemental file 3.

## Discussion

The present research showed that PVT1 is upregulated while GAS5 is downregulated in OA. In addition, PVT1 and GAS5 negatively regulate each other's expression to affect chondrocyte apoptosis induced by LPS.

PVT1 participates in cancer biology [21]. PVT1 is usually overexpressed in cancers to accelerate cancer growth and metastasis and inhibit cancer cell apoptosis [13]. A recent study reported that PVT1 sponges miR-488-3p to promote chondrocyte apoptosis [13]. We also observed an increased PVT1 accumulation in OA and its role in promoting cell apoptosis. Interestingly, LPS increases PVT1 accumulation in chondrocytes. It is known that LPS may regulate TLR4, which plays a critical role in the pathogenesis of OA [22]. Therefore, increased LPS accumulation in OA patients may upregulate PVT1, which in turn aggregates disease conditions by promoting cell apoptosis. Our finding and the previous study [18] showed that PVT1 and GAS5 might play opposite roles in cell apoptosis.

Different from the role of PVT1 in chondrocyte apoptosis, GAS5 plays a protective role in LPS-induced inflammatory injury by upregulating KLF2 to suppress cell apoptosis [14]. We observed the downregulation of GAS5 in OA and its inhibitory effects on LPS-induced apoptosis of chondrocytes. In addition, LPS treatment experiment suggested that GAS5 downregulation in OA is likely caused by increased LPS levels. Therefore, our data suggest that both PVT1 and GAS5 participate in OA through an LPS-dependent pathway. Interestingly, IL-1β treatment of chondrocytes alters PTV1 expression but not GAS5 expression (Supplemental Figure S1). A recent study also used IL-1β to simulate acute inflammatory response in chondrocytes and LPS to simulate acute exacerbation of chronic inflammatory states [23]. Local pathological process in OA cartilage leads to changes in chondrocyte phenotypes, including the production of inflammatory mediators that may be associated with chronic OA pain processes. The pain mediators in acute and chronic pain may be different, and the underlying mechanisms that lead to peripheral or centrally mediated pain may also be different [20]. In this study, PVT1 and GAS5 mainly interact with LPS. Therefore, we speculated that the interaction between PVT1 and GAS5 may affect the chronic pain of OA.

LncRNAs play their roles mainly by regulating the expression of protein-coding genes at different levels [11,12]. In certain cases, lncRNAs may also interact with miRNAs by serving as their sponges or methylation mediators [24,25]. However, the interactions between lncRNAs remain hardly known. In this study, we reported that PVT1 and GAS5 could form a negative feedback loop to regulate cell apoptosis. It has been reported that lncRNAs may bind to the promoter of certain genes to inhibit transcription [26]. For instance, CISAL can form an RNA–DNA triplex structure with the promoter of BRCA1 gene, thereby preventing the binding of BRCA1 transcription factor GABPA to the regulatory binding region [26]. We showed that PVT1 and GAS5 could directly bind to each other’s promoter regions. Our findings enriched our understanding of the functionality of lncRNAs. Because PVT1 and GAS5 are only closely correlated in OA patients, but not healthy controls, we speculated that the binding of PVT1 and GAS5 to each other’s promoters might be mediated by certain pathological factors. Recent studies have characterized the critical functions of a considerable number of lncRNAs with critical functions in OA. These lncRNAs are expected to serve as potential targets to treat OA [27,28]. However, this study only included grade 4 OA patients. In addition, the *in vivo* interaction between PVT1 and GAS5 in OA is unclear. Moreover, the downstream targets of PVT1 and GAS5 in OA are unknown. Therefore, future studies are still needed.

## Conclusion

PVT1 is upregulated, while GAS5 is downregulated in OA synovial fluid and tissue samples. PVT1 and GAS5 may form a negative feedback loop to regulate the apoptosis of chondrocytes induced by LPS.

## Supplementary Material

Supplemental Material
