# Supplementary material for: Long non-coding RNA plasmacytoma variant translocation 1 and growth arrest specific 5 regulate each other in osteoarthritis to regulate the apoptosis of chondrocytes
Source: Bioengineered. 2022 Jun 15;13(5):13680–8. doi: 10.1080/21655979.2022.2063653 (PMC9275885; doi:10.1080/21655979.2022.2063653)
Supplement: Supplemental Material [file KBIE_A_2063653_SM5769.zip › supplemental file 1 ethical approval .pdf]

The Ethics Committee of

the Second Affiliated Hospital of Fujian Medical University Approval

Principal Investigators: Liquan Cai, Nianlai Huang, Xiaolu Zhang, Shiqiang Wu, Liangming Wang, Qingfeng Ke

Title of Projects: The investigation of PVT1 in osteoarthritis

Date Submitted: May 18, 2017

Date Approved: May 20, 2017

The Ethics Committee of the Second Affiliated Hospital of Fujian Medical University has reviewed the proposed use of human subjects in the above-mentioned projects. It is recognized that the rights and the welfare of the subjects are adequately protected; the potential risks are outweighed by potential benefits. We approve papers resulting from the project.

刘光煜

The Ethics Committee of the Second Affiliated Hospital of Fujian Medical University

May 20, 2017
