# Supplementary figures and images for: Long non-coding RNA plasmacytoma variant translocation 1 and growth arrest specific 5 regulate each other in osteoarthritis to regulate the apoptosis of chondrocytes
Source: Bioengineered. 2022 Jun 15;13(5):13680–8. doi: 10.1080/21655979.2022.2063653 (PMC9275885; doi:10.1080/21655979.2022.2063653)

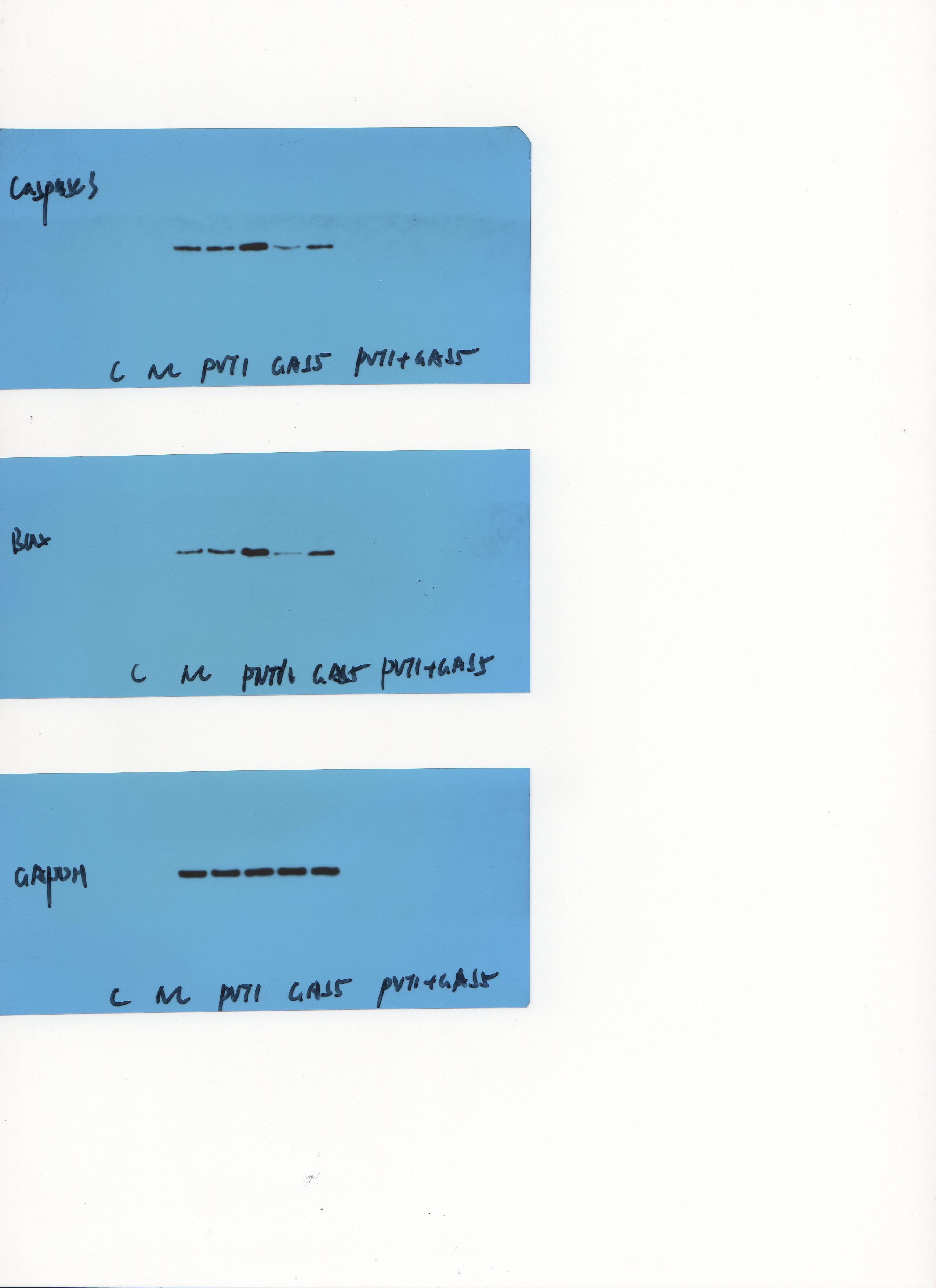

Supplement: Supplemental Material [file KBIE_A_2063653_SM5769.zip › supplemental file 3 original image for figure 6 Cas3GAPDH (1).tif]

GAS5-100X1


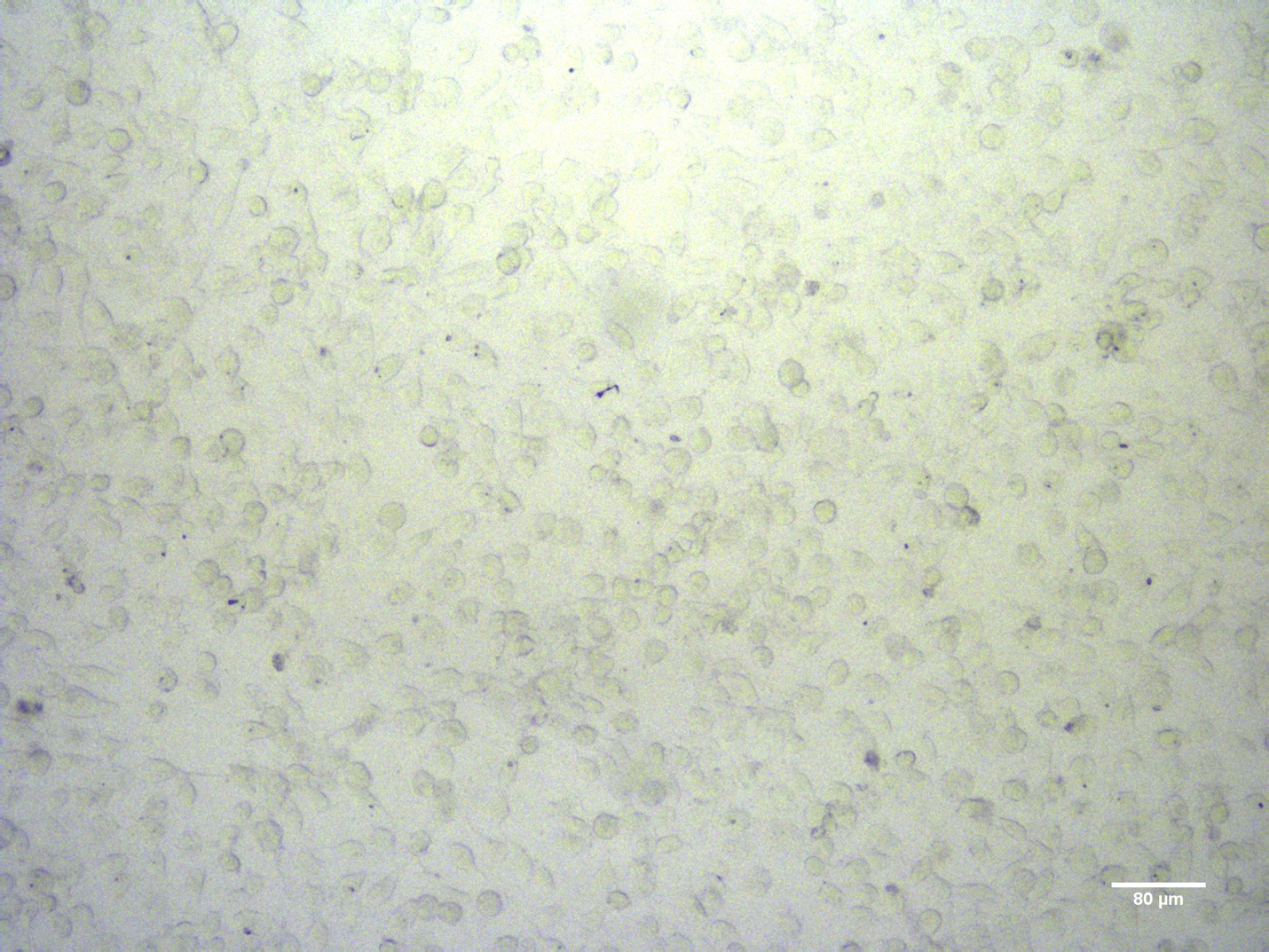


GAS5-100X2


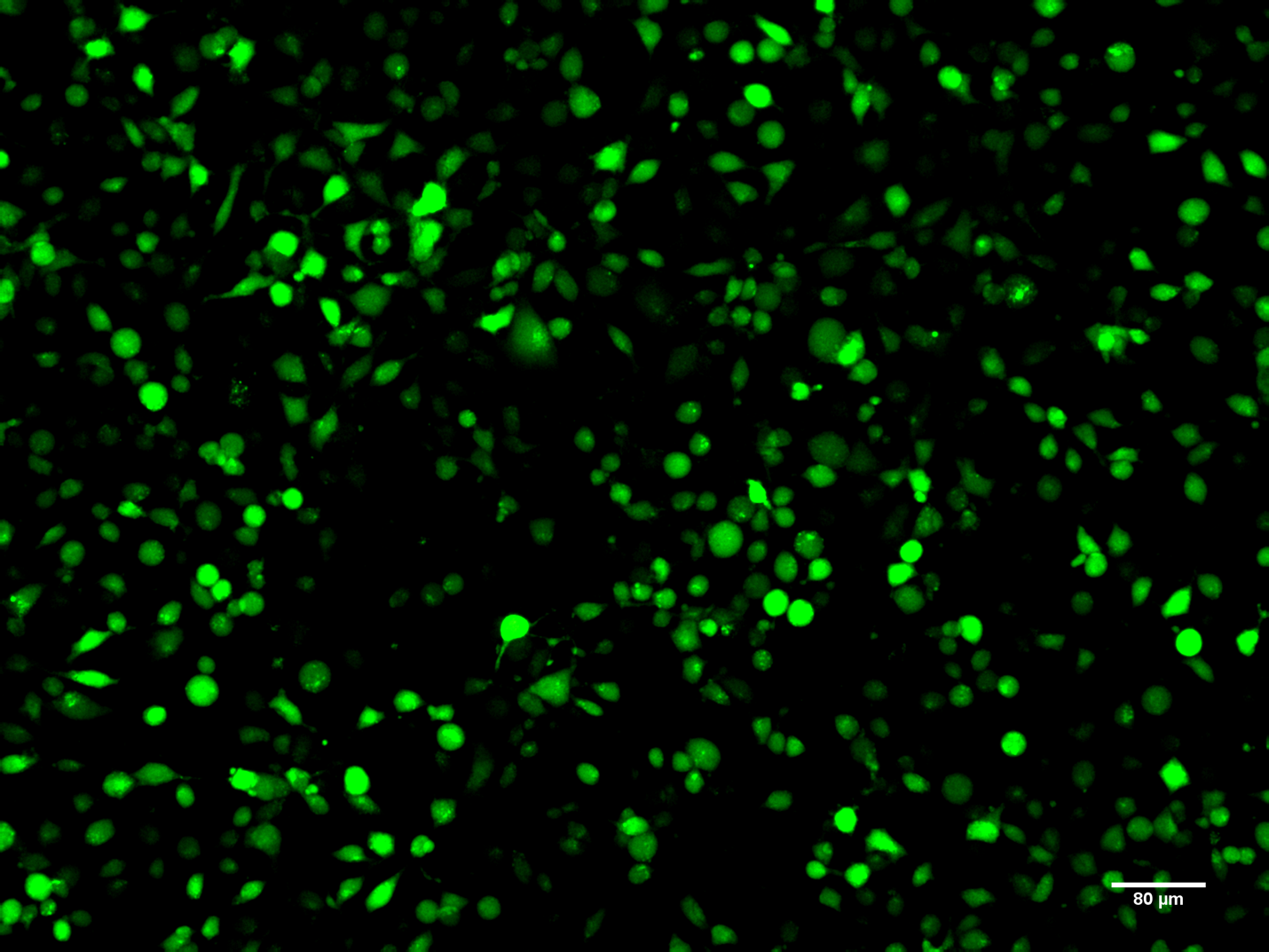


GAS5-100-merge


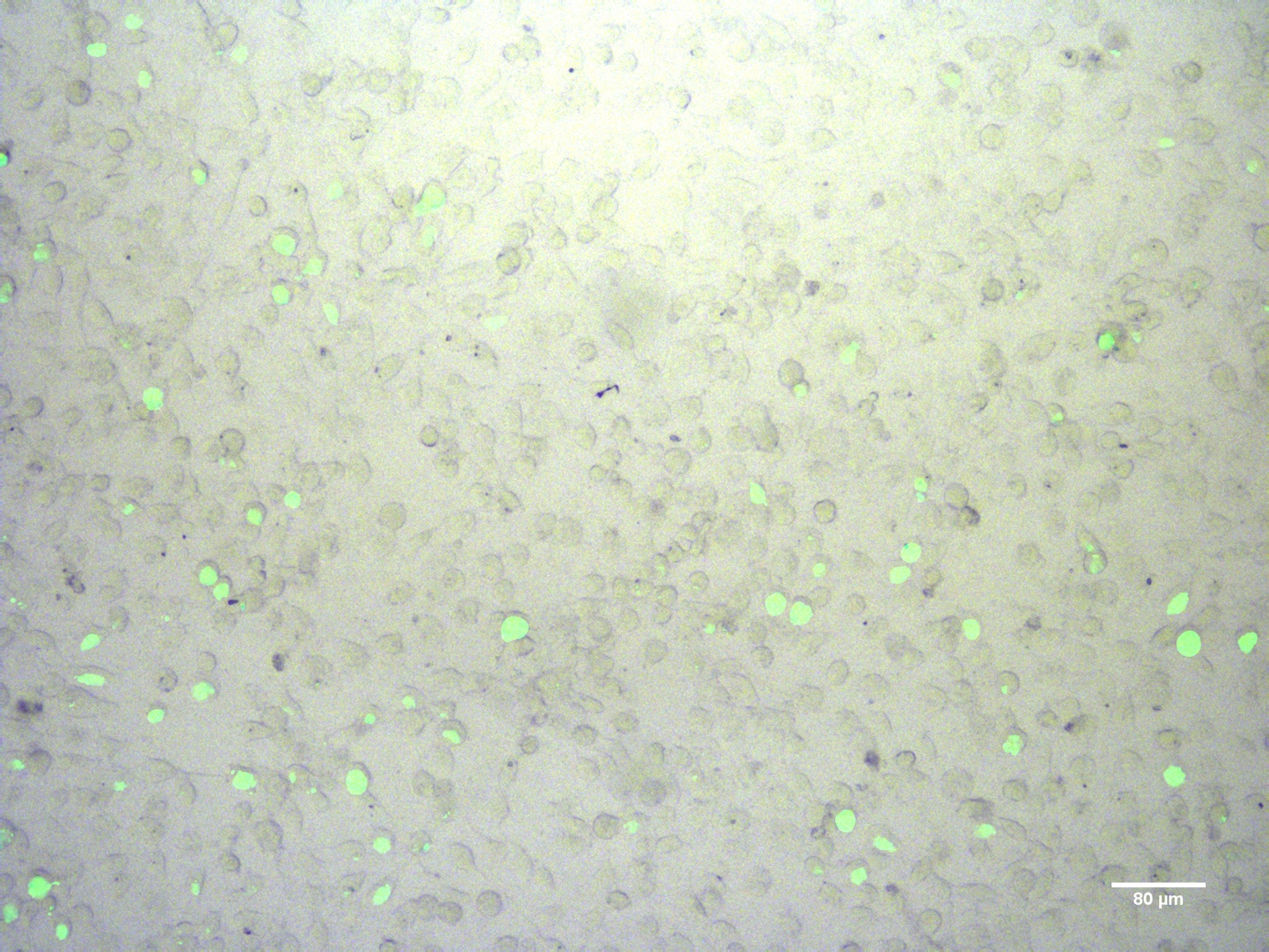


PVT1-100X1


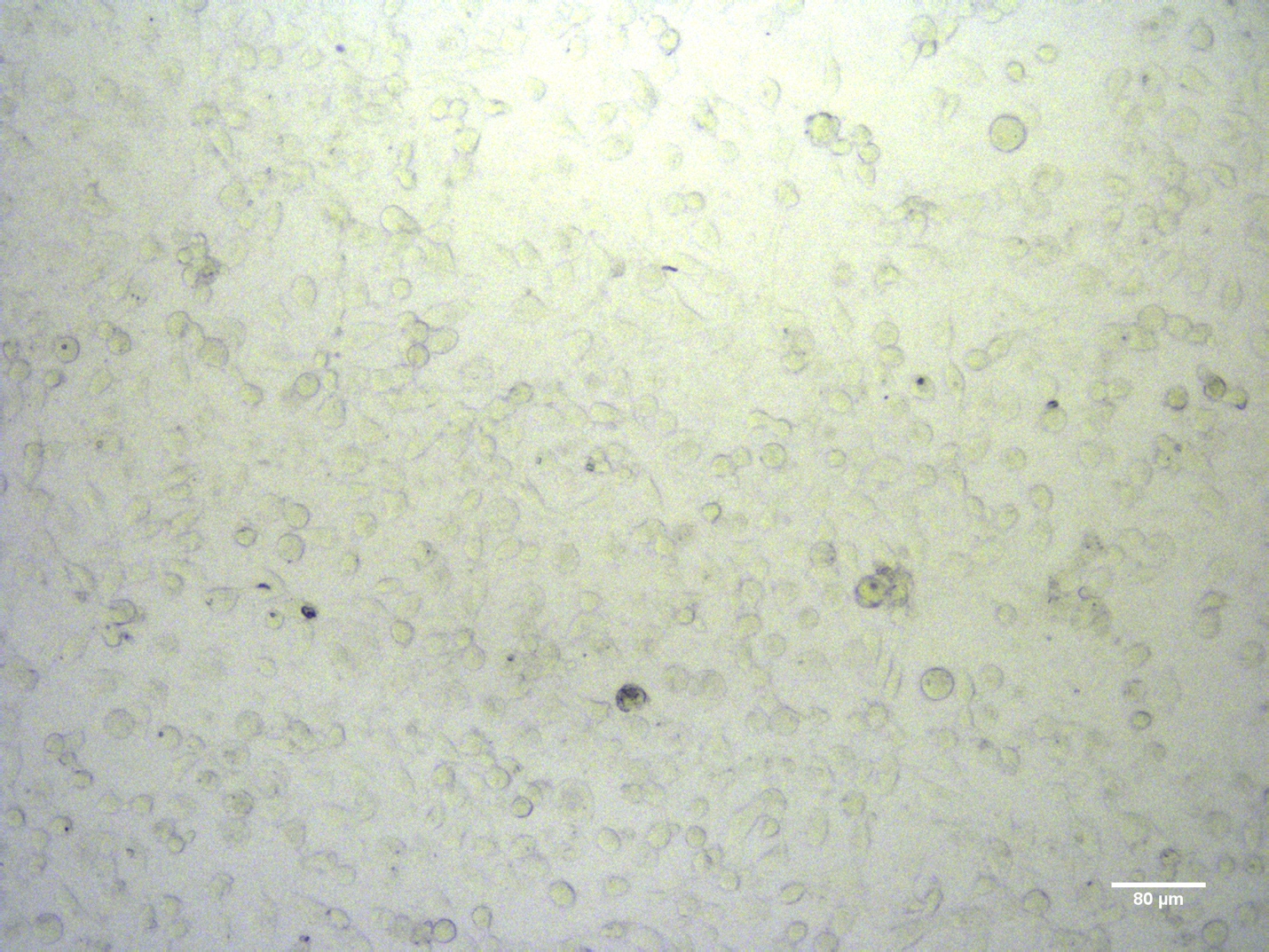


PVT1-100X2


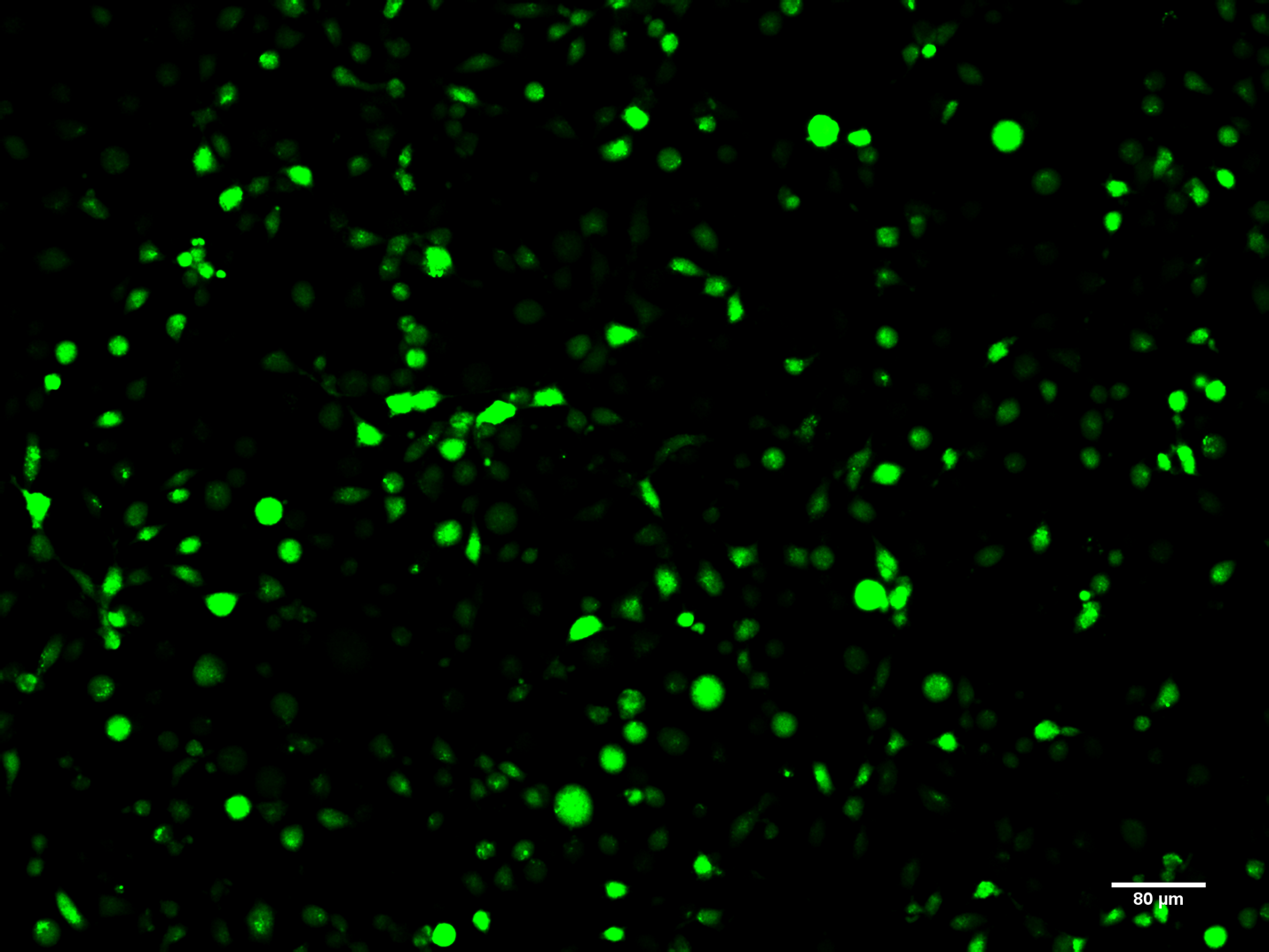


PVT1-100-merge


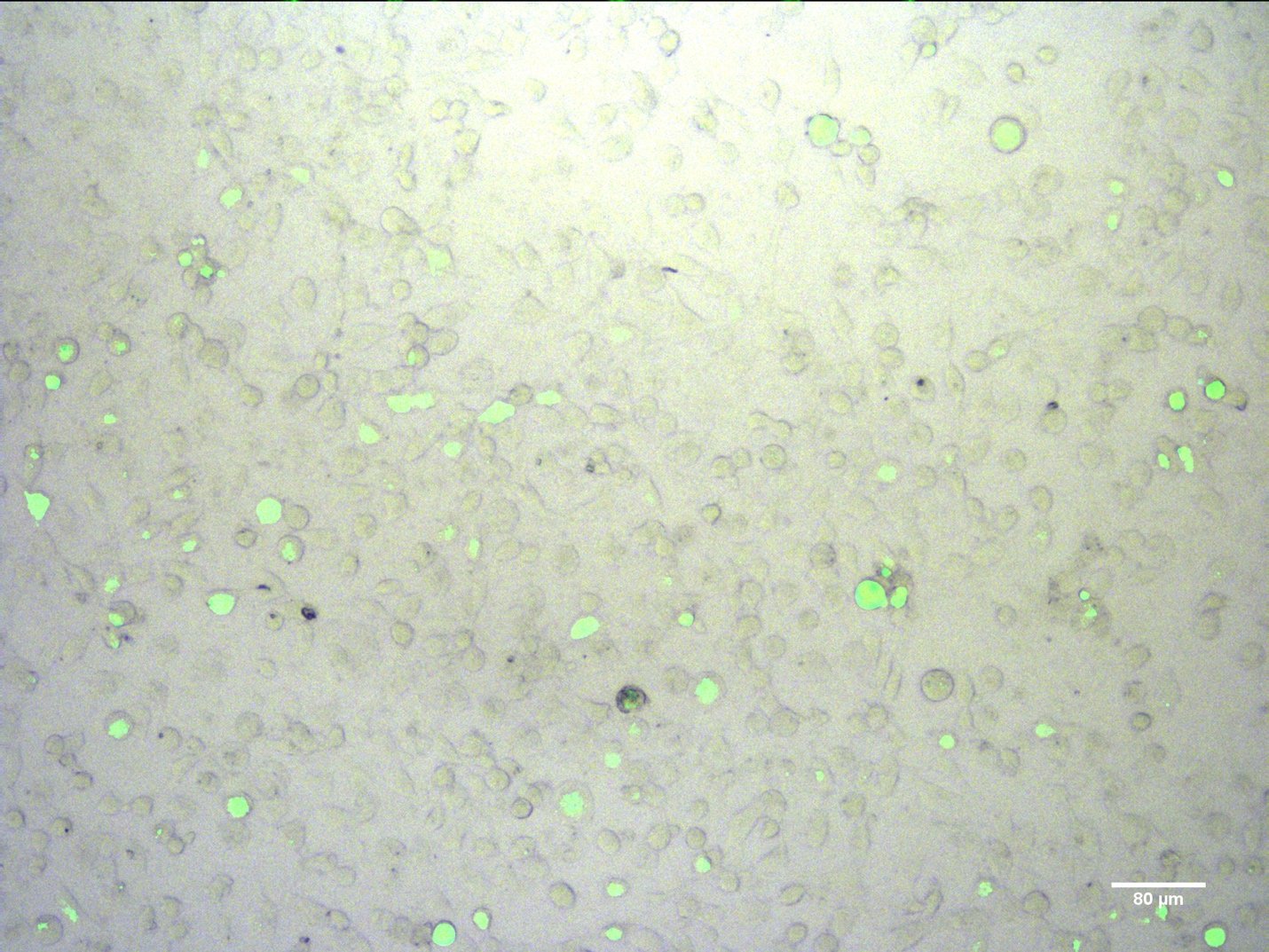

Supplement: Supplemental Material [file KBIE_A_2063653_SM5769.zip › supplemental file 2.docx]

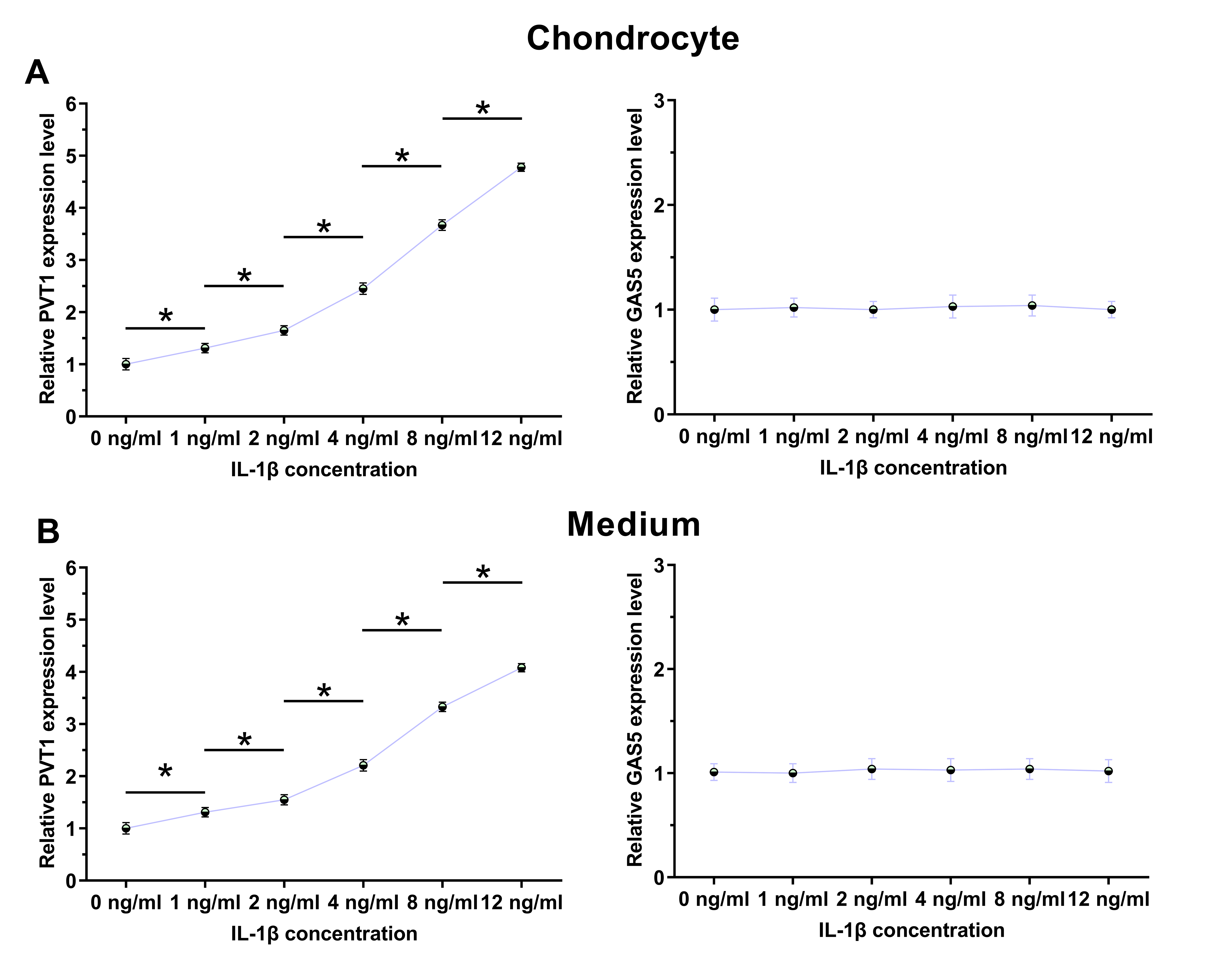

Supplement: Supplemental Material [file KBIE_A_2063653_SM5769.zip › Revised Supplemental figure 1.tif]
